# A study of Aspergillus niger- hydrolyzed cassava peel meal as a carbohydrate source on the histology of broiler chickens

**DOI:** 10.1186/2193-1801-3-31

**Published:** 2014-01-17

**Authors:** Adeyemi Isaiah Adeyemo, Alhassan Sani, Temitayo Abosede Aderibigbe, Muhammed Ola Abdurrasheed, James Oludare Agbolade

**Affiliations:** Biological sciences Department, Ondo State University of Science and Technology, Okitipupa, Ondo State Nigeria; Department of Microbiology, University of Ilorin, Ilorin, Kwara State Nigeria; Department of Animal production, University of Ilorin, Ilorin, Kwara State Nigeria; Histopathology Unit, University of Ilorin Teaching Hospital, Ilorin, Kwara State Nigeria; Plant Biology and Biotechnology Department, Federal University of Oye Ekiti, Oye-Ekiti, Ekiti State Nigeria

**Keywords:** Hydrolyzed cassava peel, Internal organs, Treatment group, Histology, Chickens

## Abstract

The purpose of this study is to investigate the effect of hydrolysed cassava peel inclusion as a replacement for maize in broiler chicken feedstuff on the histology of the internal organs of broiler chickens. Thirty six, two weeks old unsexed broiler chickens were used for the study in a feeding trial of forty two days. The chickens were randomly allocated to six dietary treatments A - F using a completely randomized design. Each treatment group contained two replicates of three broiler chickens. Group A chickens (A1 and A2) were fed with the control diet (0% hydrolyzed cassava peel as main carbon source). Groups B-E (in replicates 1 and 2) were administered with experimental diets containing 25%, 50%, 75%, 100% of hydrolyzed cassava peels respectively replacing maize while group F (F1 and F2) were fed with diet containing 100% unhydrolyzed cassava peels replacing maize as the main carbon source. Feed and water were supplied ad libitum for the six weeks feeding trials period. Vaccine and drugs were administered as at when due. At the end of the third week, two replicate per group were fasted for twelve hours and slaughtered. Samples of liver, kidney and heart were collected and tissue samples were taken for histological examinations. All the chickens in group F that fed on unhydrolyzed cassava peel recorded 100% mortality within the first six days of the feeding trials while those in groups A to E recorded 0% mortality. Histology of the kidney, heart and liver showed increasing mark of coagulative necrosis, degeneration of the hepatocytes and vacuolations due to the shrinking of the hepatocellular and cardiac tissues as the cassava inclusion level increases in feed. It is concluded that birds can be fed with maize replaced with up to 50% hydrolyzed cassava peel in chicken feeds without serious deleterious effects and that the wastes have useful products in animal nutrition. Also, the replacement added economic in chicken production. The hydrolysis has led to a reduction in the potency of cyanide in the peel thereby making it a safe and possible candidate in the production of chicken feeds.

## Introduction

The world shortage of cereals global inflationary trends coupled with the use of maize as raw material for ethanol production in the vulnerable bio- fuel industry around the world necessitates the development of livestock feeding systems which are independent of maize and other cereals (Moo-young et al. 1987; Christopher –Berg 2006; Kayode 2009 FIDAAfrique- IFADAfrica 2010). It is for this reason that in Nigeria, recent emphasis is placed on “backward integration” a design by the federal government to encourage industrialists, food scientists, livestock farmers and manufacturers to rely on local sources of raw materials which has previously depended on importation (Sani et al. 1992; Aletor 1990; Kayode 2009; Adeyemo 2003). That food scarcity is a plague in many developing countries of the world, including Nigeria where daily intake of animal protein per caput falls far below the normal intake as recommended by FAO (1986), is not in doubt. To alleviate this situation, it has been realized that broiler production is the fastest and easiest route (Nworgu *et al.*^2000^; Dipeolu *et al.*^1996^; Larry 1993) since they are prolific, possess a high feed conversion ratio and are accepted by all, irrespective of religion. However, feed cost are presently very high and makes up to 60-70% (Larry 1993) or 70-80% (Oruwari *et al.*^1995^*)* of the total cost of production in Nigeria compared to 50-70% in developed countries (Thackie and Flenscher 1995). This therefore highlights the importance of feed management to broiler producers.

Thus it is necessary to reduce the cost of feeds in order to produce cheaper products without affecting profits. Since energy source constitutes 45-60% of finished feeds for monogastric animals (Steiner et al. 1994) and birds eat to satisfy their energy requirement (Sibbald 1982), cassava peels which is available in large quantity is being investigated to serve as alternative main energy source in broiler feedstuffs (Belewu and Banjo 1999; Hafeni et al. 2013; Sabiiti 2011). Although several authors have reported suboptimal performance or death when monogastrics are fed cassava peel based diets claiming these are due to the cyanide content of the cassava peel. However, little attention has been given to the evaluation of the effects of intake of residual cyanide in processed cassava peel on the histology of internal organs of broiler chickens. This study was therefore designed to address this issue.

## Materials and methods

Cassava peel was collected from from five agricultural waste dumpsites in Ilorin metropolis, Kwara state. They were then dried in an oven at 80°C and then ground into very fine powder. The ground powder was dried into constant weight in an oven at 60°C and their proximate analyses determined by AOAC 2000 method. Cyanide content was determined using the modified method of picrate paper kit as developed by Bradbury *et al.* (1999). The grounded powder was stored in a desiccator until required.

### Source and maintenance of organisms

Five grams of the freshly obtained peel was taken from the sealed cellophane bags (with which they were collected from source) into sterile universal bottles containing 100 ml of distilled water and shaken thoroughly. The settled supernatants were decanted off into separate test tubes serving as stock solutions. Serial dilutions of the stock were made with 0.1 ml of the aliquot spread on Potato Dextrose Agar (PDA) figures containing 1% streptomycin. (to inhibit bacterial growth). The figures were incubated at room temperature (27 – 31°C) for 48 hours (Lawal *et a*l. 2005; Berghem et al. 1976; Chahal 1992, Bukoye 2001). After incubation, representative fungal colonies were picked from each figure and purified on fresh PDA figures. The purified isolates were transferred to PDA slants incubated at room temperature for 48 hours and stored at 4°C. The isolates were identified according to the scheme of McGinnins (1980).

### Screening for cellulose Production

The method of Bisaria and Ghose (1981) was used. Point inoculations of the spores of the isolates were grown on PDA supplemented with Carboxylmethylcellulase (CMC, 2% W/V) medium. The figures were incubated at room temperature (27°C- 32) for 72 hours after which it was stained with 2% Congo red solution for 15 minutes. Excess dye was removed by washing with 1 M NaCl and the figures were fixed with 1 M HCl. The production of extracellular cellulase by the organism was indicated by a zone of clearance around the fungal colonies on the figure. The zone of clearance was measured on each figure and the average determined. *Aspergillus niger* AC4 showing the biggest zone of clearance was selected for further studies.

### Production of crude cellulase

Mineral salts media (MSM) for cultivation of fungal isolates was prepared with compositions as shown below {g/l}. KH_2_PO _4_, 10 g; (NH4)_2_SO4, 10.5 g; MgSO_4_.7H_2_O, 0.3 g; CaCl_2_, 0.5 g; FeSo_4_, 0.013 g; MnSO_4_.H_2_O 0.04; ZnSO_4_.7H_2_O 0.04; Yeast extract 0.5 g; Carbon source 40 g( The carbon sources were delignified cassava peel and carboxylmethylcellulase). One hundred and fifty millilitre of each medium was dispersed into conical flask and sterilized in the autoclave at 121°C for 15 minutes. The final pHs of the medium was adjusted to 5.0 with 0.1 M NaOH and 0.1 M HCl using a pH meter (pyeunicam pH).Spores of 72 hour old cultures of *Aspergillus niger* AC4 were harvested by washing slants with 10 ml of sterile distilled water. An aliquot of 5 ml of the spore suspension was used to inoculate 150 ml of each of the prepared medium. The culture media were incubated at room temperature (27-32°C) in an orbital shaker (Gallenhamp, England) at 100 rpm for seven days.

### Hydrolysis of cassava peel and CMC

The ability of the crude enzyme to hydrolyze cassava peel was studied using the grounded cassava peel sample while commercial carboxymethylcellulase (CMC) was used as the standard. Cellulase activity of the suction culture filtrate was determined colorimetrically by measuring the increase in reducing groups by the hydrolysis of Carboxylmethylcellulase (CMC) substrate and the cassava peel (Ali *et al*. 1991; Panda 1989). Cultured samples were filtered through whatmann filter paper to remove the mycelia and other particle. The filtrates were used to assay enzymatic activity. The reaction mixture containing 0.5 ml of the enzyme solutions and 1 ml of 2% (w/v) of the carbon sources were incubated at a temperature of 35°C for 20 minutes and the reactions stopped by adding 2.0 ml of DNS Reagent materials (1.0 g of 3,5-dinitrosalicylic acid, 20 ml of NaOH, 30 g of sodium potassium tartarate in 100 ml). The total mixture was then heated for 5 minutes, cooled and 20 ml of distilled water added. The colour intensity was determined at 560 nm using a spectrophotometer (Jenwey 6405 UV/visible). Cassava peel sample was found to have a cellulase activity of 240 U/ml while CMC gave a cellulase activity of 54 U/ml.

### Experimental diets

The delignified hydrolyzed cassava peel and other ingredients were used to formulate starter and finisher diets respectively at 25%, 50%, 75% and 100% replacement value for maize to meet the NRC (1984) nutrient requirement of broiler chickens. The feed containing 100% maize was used as control. Soya bean oil was added to obtain equal metabolizable energy.

### Experimental birds

A total of thirty two weeks old unsexed broiler chickens with average initial bodyweight of 0.685 ± 0.0027 g were divided into six (6) groups. They were bred and maintained in a six partitioned clean cage, with well-ventilated conditions (temperature 27 ± 5°C; photoperiod: 12 h natural light and 12 h dark; humidity: 45-50%). Cleaning of the cages was done daily. They were kept under this condition for the period of the experiment. The chickens were randomly allocated to six dietary treatments A - F using a completely randomized design. Group A chickens were fed with the control diet (0% hydrolyzed cassava peel as main carbon source). Groups B-E were administered with experimental diets containing 25%, 50%, 75%, 100% of hydrolyzed cassava peels respectively replacing maize as energy source while group F was fed with diet containing 100% unhydrolyzed cassava peels replacing maize as the main carbon source. Feed and water were supplied ad-libitum for the six weeks feeding trials period. Vaccine and drugs were administered as at when due.

### Measurements

The study lasted for 42 days. At the end of the third week of the feeding trails, 2 birds per replicate were randomly selected, fasted for 12 hours to empty their gastrointestinal tract, weighed individually, slaughtered, and eviscerated. For histological analysis, tissue samples of each organ were taken, immersed in 10% formolsaline for 72 hours, and processed for paraffin embedding, using ethanol for dehydration and xylene as clearing agent. Sections from each organ were made at a thickness of 4 μm with a Leica rotary microtome, stained with hematoxylin-eosin, and examined by light microscope.

### Data analysis

The data obtained were subjected to analysis of variance and the means were compared using the Duncan Multiple range test. A significant level of 0.05 was used. The experiments were all designed as a complete randomized design (CRD).

## Result and discussion

Table 1 shows the proximate analysis of the cassava peel used during the period of the experiment. The result showed that cassava peel is very rich in carbohydrate content but very low in protein content as earlier reported by Tewe and Egbunike (2007). It also has a very high digestible energy but low in crude fibre. It has a cyanide content of 0.74 mg/100 g of cassava peel but after hydrolysis by the culture filtrate of the *Aspergillus niger* AC4, cyanide content reduced to 0.08 mg/100 ml. Table 2 shows the result of the proximate composition of the experimental feeds for each group A- E (A^fe^ to E^fe^) and feaces from the birds in each group (A^fc^ to E^fc^) for the five experimental chickens fed 0%, 25%, 50%, 75% and 100% in groups A- E respectively. There is increase in crude protein of faeces compared to the feed because of the presence of uric acid, the excretory product of the chickens that is egested with the faeces while there is a reduction in crude fibre a cellulolytic material because of its hydrolysis by the incorporated cellulase enzyme. There is a reduction in total ash of faeces compared to the feed because the chickens have made use of part of the mineral content of the feed. Crude fat increases because it has become concentrated in faeces since the faeces has absorbed more water as evident by the moisture content of faeces being higher than that of diets. All the chickens in group F that fed on unhydrolyzed cassava peel died within the first six days of the feeding trials. Protein retention of broilers also decreased with increase in cassava inclusion level but was not significantly different until inclusion level beyond 50%. the kidney and liver were moderately damaged such that their functions were hampered and some essential nutrients which should have been used for body building of the chickens were now passed out with the faeces hence the increase in protein content of the faeces compared to diets. This is similar to the result obtained by Muhammad and Oloyede 2009.

**Table 1:** **Proximate analysis of the cassava peel**

Carbon source	Dry matter (%)	Crude protein (%)	Carbo - hydrate (%)	Crude fat (%)	Crude fibre (%)	pH	Total ash (%)	Moisture content (%)	Nitrogen free extract (NFE)	Gross energy MJ/Kg	Digestible energy MJ/Kg	Cyanide mg/100 g
**Cassava peel**	**90.94**	**4.15**	**93.5**	**0.84**	**3.35**	**5.7**	**4.37**	**9.06**	**67.70**	**1.65**	**1.03**	**0.74**

**Table 2:** **Proximate analysis of feeds and faeces ( Fed and egested respectively ) by the experimental chickens**

Bird group	Moisture content (%)		Dry matter (%)		Crude protein (%)		Crude fat (%)		Crude fibre (%)		Total ash (%)		Cyanide (mg/100 g)	
	Feed (fe)	Faeces (fc)	Feed (fe)	Faeces (fc)	Feed (fe)	Faeces (fc)	Feed (fe)	Faeces (fc)	Feed (fe)	Faeces (fc)	Feed (fe)	Faeces (fc)	Feed (fe)	Faeces (fc)
A	9.37^b^	11.84^a^	90.63^b^	88.16^a^	17.06^d^	22.09^a^	3.67^ac^	4.24 ^b^	9.95^ab^	5.04^a^	25.06^a^	13.33^a^	0.08^a^	0.08^a^
B	7.74^c^	11.52^a^	92.26^a^	88.48^a^	15.09^c^	19.25^b^	3.24^c^	4.52^b^	9.50^a^	7.60^bc^	30.24^c^	14.86^b^	0.08^a^	0.08^a^
C	10.26^a^	11.20^a^	89.74^ab^	88.80^a^	14.43^bc^	17.50^c^	2.15^b^	3.76^ab^	9.55^a^	7.87^bc^	26.73^a^	20.96^c^	0.08^a^	0.08^a^
D	9.18^b^	12.38^b^	90.82^b^	87.62^b^	11.59^a^	18.59^bc^	2.67^bc^	3.78^ab^	11.19^b^	6.47^b^	30.44^c^	21.24^bc^	0.08^a^	0.08^a^
E	7.82^c^	11.24^a^	92.81^a^	88.76^a^	13.78^b^	17.50^c^	1.35^a^	3.37^a^	13.30^c^	7.92^c^	29.27^b^	23.44^d^	0.08^a^	0.08^a^

^abcd^Means with different superscripts in a column are significantly different (p < 0.05).

Values are means of three replicate determinations.

**Figure 1:**
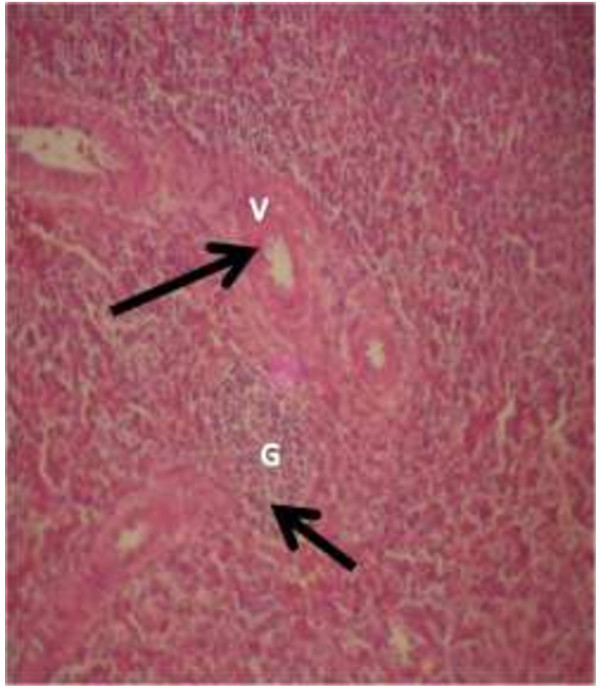
**Photomicrograph of kidney of chickens that fed on Hydrolyzed cassava peel at 0% as main energy source.**

**Figure 2:**
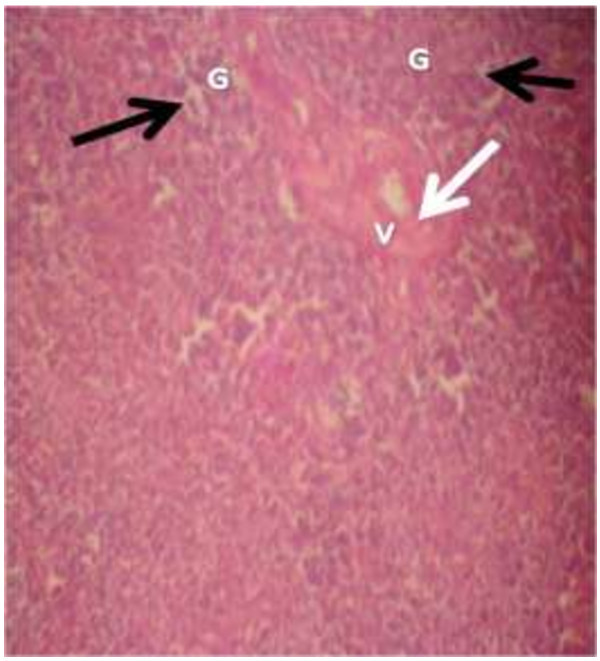
**Photomicrograph of kidney of chickens that fed on Hydrolyzed cassava peel at 25% as main energy source.**

**Figure 3:**
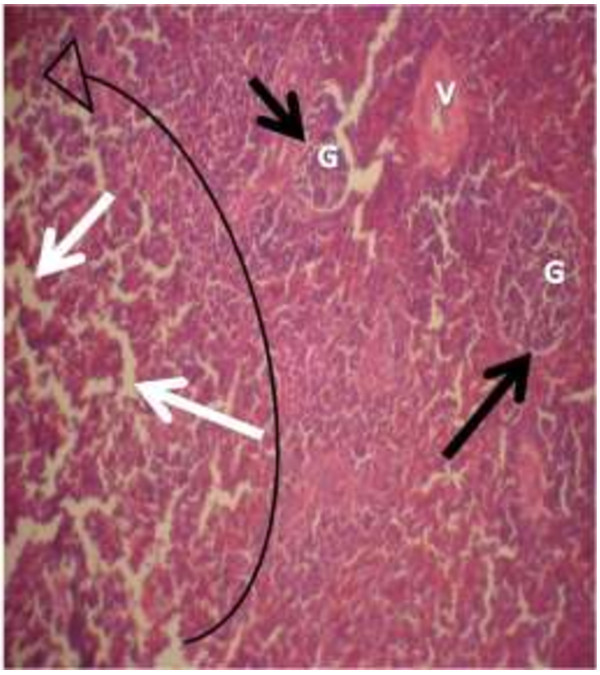
**Photomicrograph of kidney of chickens that fed on Hydrolyzed cassava peel at 50% as main energy source.**

**Figure 4:**
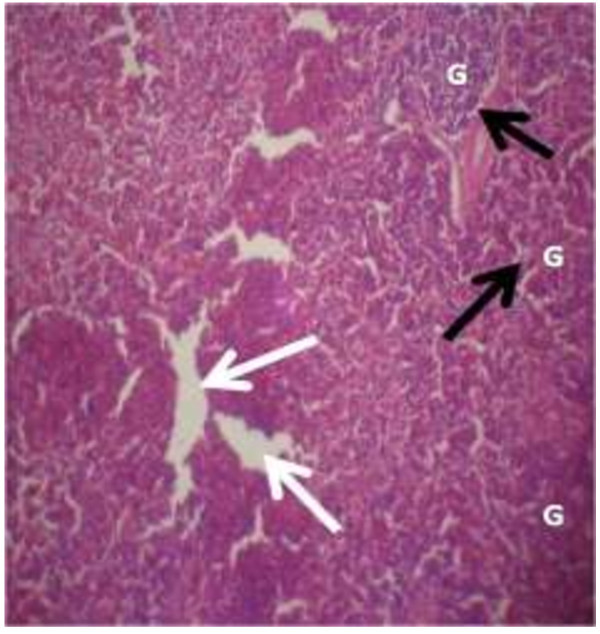
**Photomicrograph of kidney of chickens that fed on Hydrolyzed cassava peel at 75% as main energy source.**

**Figure 5:**
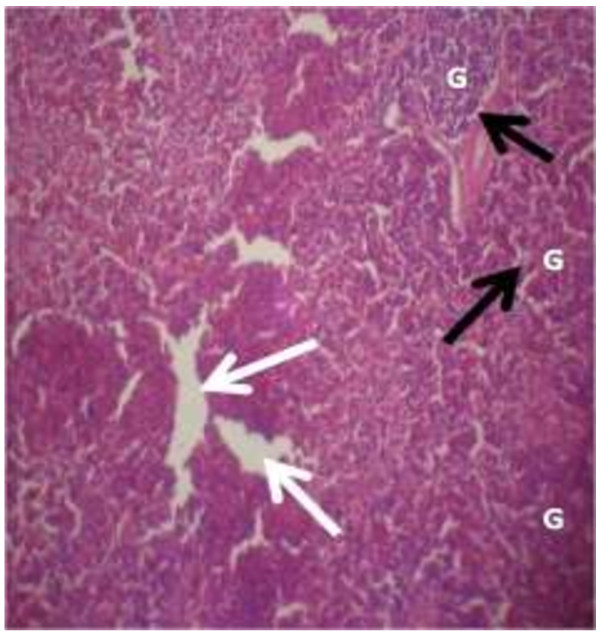
**Photomicrograph of kidney of chickens that fed on Hydrolyzed cassava peel at 100% as main energy source.**

**Figure 6:**
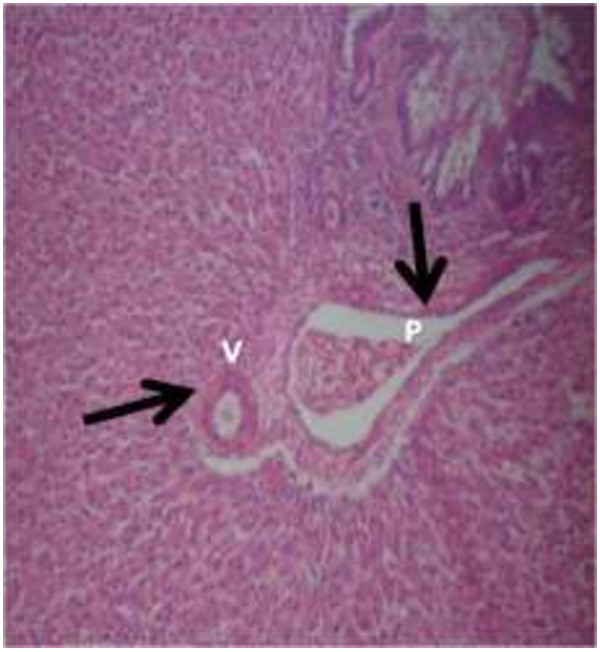
**Photomicrograph of Liver of chickens that fed on Hydrolyzed cassava peel at 0% as main energy source.**

**Figure 7:**
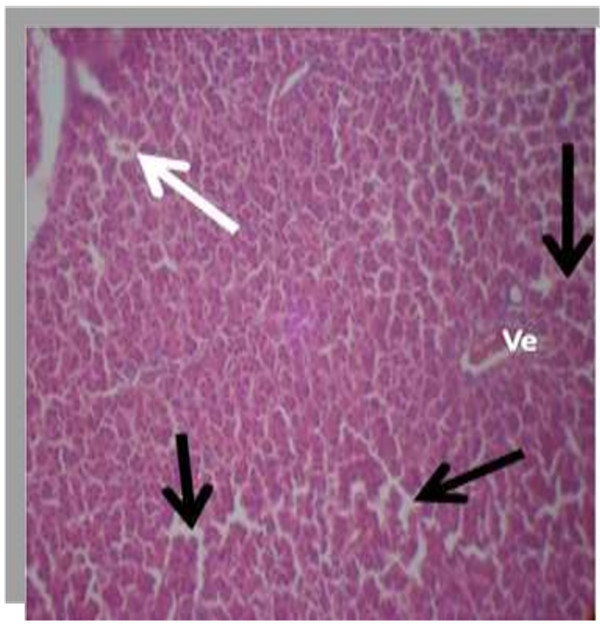
**Photomicrograph of Liver of chickens that fed on Hydrolyzed cassava peel at 25% as main energy source.**

**Figure 8:**
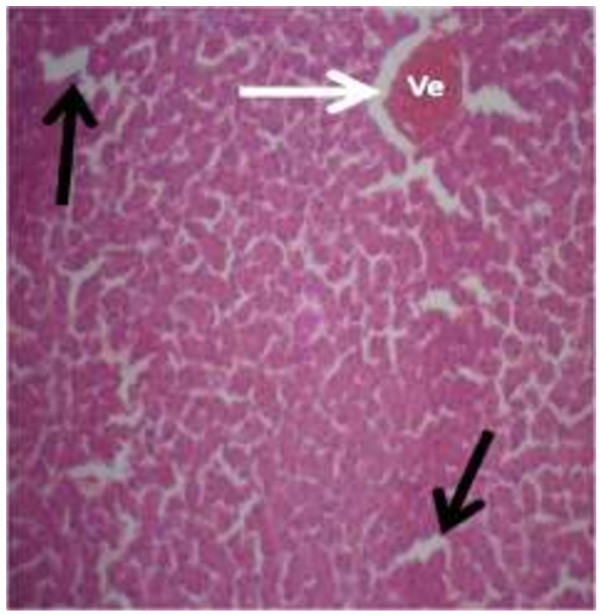
**Photomicrograph of Liver of chickens that fed on Hydrolyzed cassava peel at 50% as main energy source.**

**Figure 9:**
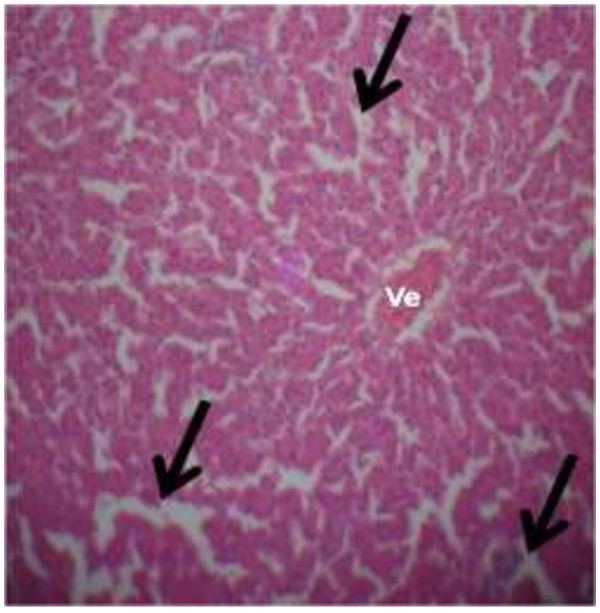
**Photomicrograph of Liver of chickens that fed on Hydrolyzed cassava peel at 75% as main energy source.**

**Figure 10:**
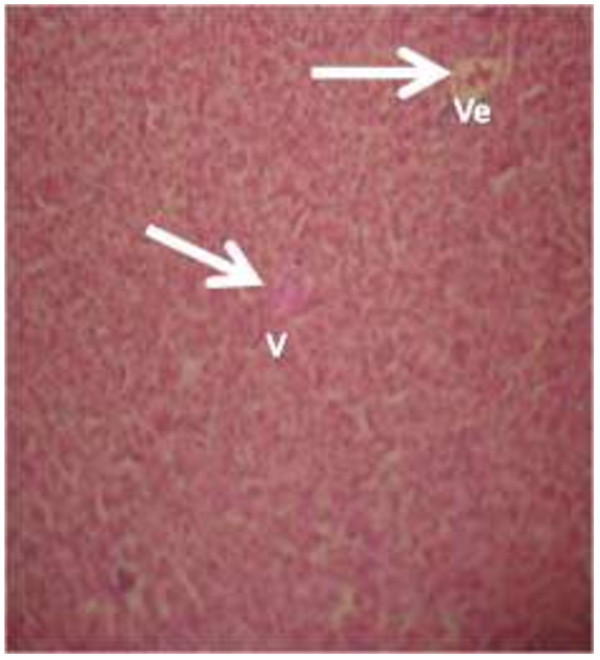
**Photomicrograph of Liver of chickens that fed on Hydrolyzed cassava peel at 100% as main energy source.**

**Figure 11:**
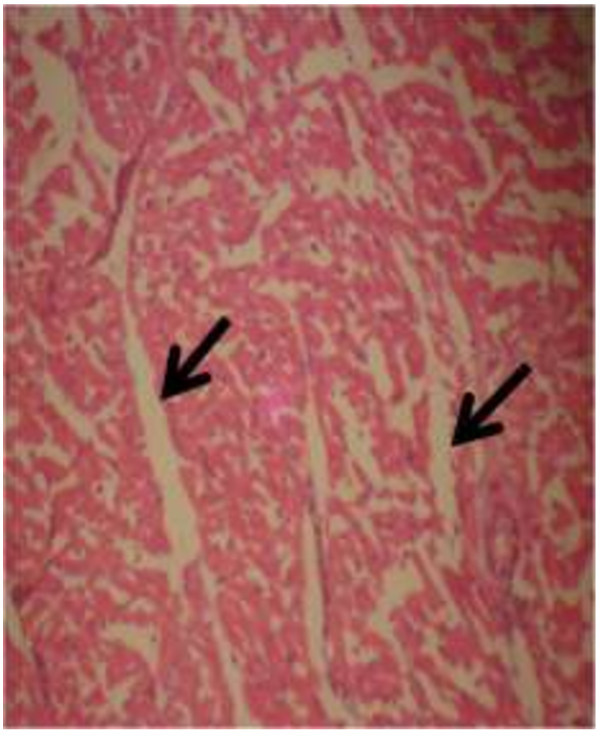
**Photomicrograph of Heart of chickens that fed on Hydrolyzed cassava peel at 0% as main energy source.**

**Figure 12:**
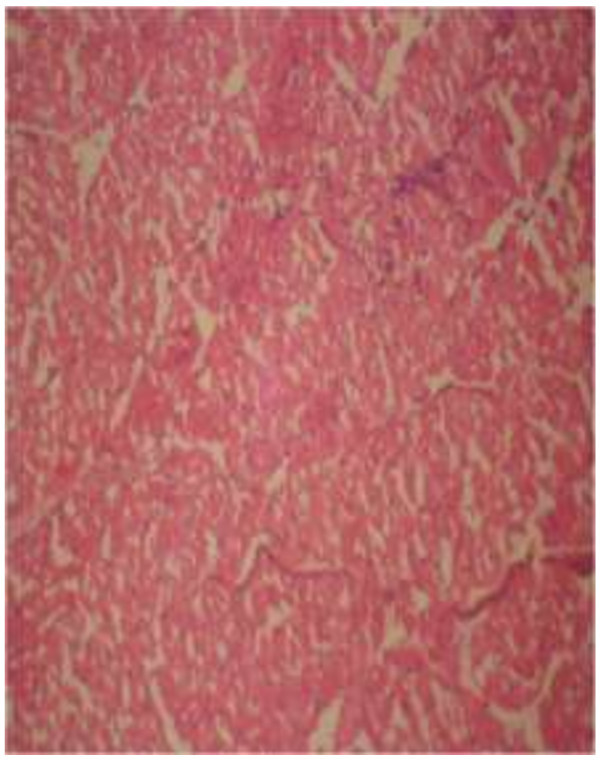
**Photomicrograph of Heart of chickens that fed on Hydrolyzed cassava peel at 25% as main energy source.**

**Figure 13:**
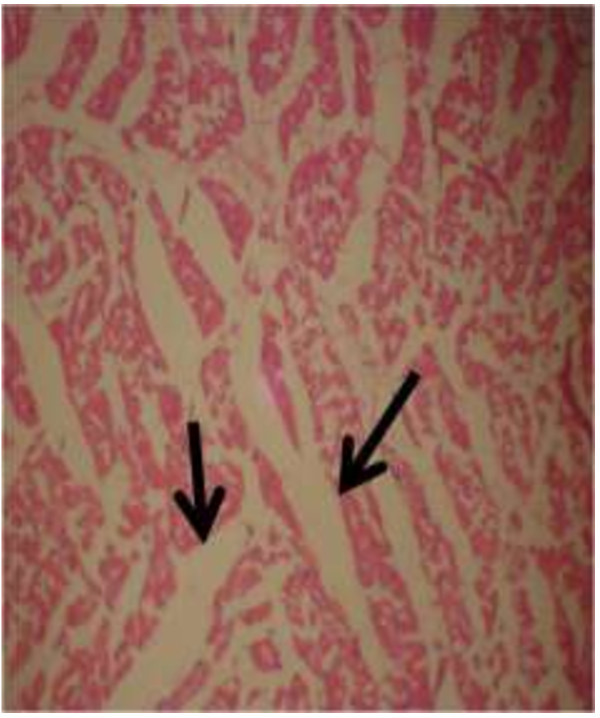
**Photomicrograph of Heart of chickens that fed on Hydrolyzed cassava peel at 50% as main energy source.**

**Figure 14:**
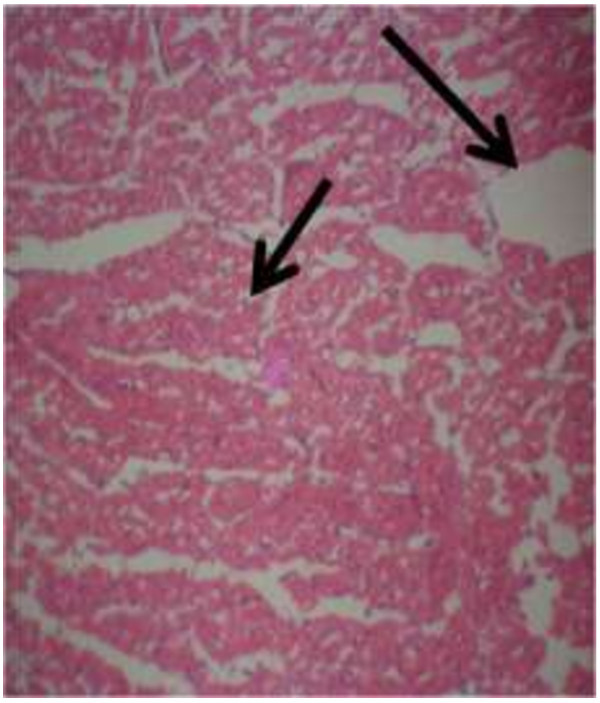
**Photomicrograph of Heart of chickens that fed on Hydrolyzed cassava peel at 75% as main energy source.**

**Figure 15:**
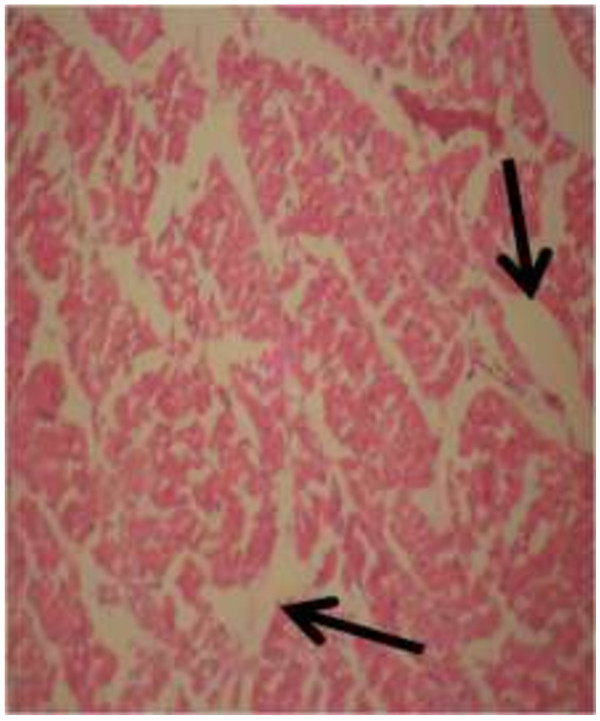
**Photomicrograph of Heart of chickens that fed on Hydrolyzed cassava peel at 100% as main energy source.**

The photomicrograph of the organs (Kidney, Liver and the Heart) of the control birds and those fed on experimental feeds 0%, 25%, 50%, 75% and 100% for groups A to E respectively are as shown on pages 10–12. Group A chickens were fed control diets while Groups B – E were fed experimental diets respectively. The kidneys of the birds fed with control diet in A were normal with black arrows showing glomerulus and vein as shown on Figure 1 while photomicrographs of Figures 2 and 3 reveals normochromic, mildly degenerated, hypercytic kidney tissues with black arrows showing degenerating glomeruli while white arrows are showing vacuolations due to the shrinking of the internal organelles. Figures 4 and 5 show degenerating glomeruli in hyperchromic, mildly degenerated, hypercytic kidney tissue with white arrows showing more visible vacuolations and black arrows showing degenerating glomeruli. Photomicrograph of the liver in Group A chickens revealed normal hepatocellular tissue with radiating hepatic cords and intact intestinal mucosa, muscle wall and serosa without any visible damage (Figure 6). Figures 7, 8, 9 and 10 however reveal mildly to moderately degenerating hepatocellular tissues with black arrows showing vacuolations while white arrows show the degenerating cells. Figure 11 shows the photomicrograph of the heart of chicken fed control diet A with normal cardiac muscle. Figures 12, 13, 14 and 15 show the photomicrographs of the heart of chickens fed experimental diets in groups B to E respectively revealing normochromic, normocytic tissue (Figures 12 and 13) and hyperchromic mildly degenerated cardiac tissues (Figures 14 and 15). The arrows indicate more visible vacuolations in the heart tissues.

In conclusion, cassava peel which is readily available in large quantity and often treated as waste could be used to replace maize which is in high demand by microbial enzymes to enhance food (protein) production particularly in developing nations like Nigeria. This would strengthen food security and provide needed protein for man.

### Compliance with ethical standards

All animal studies have been approved by the appropriate ethics committee and have therefore been performed in accordance with the ethical standards laid down in the 1964 Declaration of Helsinki and its later amendments.

## References

[CR1] Adeyemo IA (2003). Studies on degradation of waste papers using microflora isolated from refuse dumpsites.

[CR2] Aletor VA (1990). Aflatoxin contamination in some Nigerian feeds and feeding stuffs: Highlight of some nutritional, physiological and economic implications. Food Chem.

[CR3] Ali S, Sayed A, Sarker RT, Alam R (1991). Factors affecting cellulase production by Aspergillu sterresusing water hyacinth. World J Microbiol Biotechnol.

[CR4] (2000). Official method of analysis.

[CR5] Belewu GI, Banjo NO (1999). Biodegradation of rice husk and sorghum stover by edible mushroom (Pleurotussajo caju). Trop J Anim Sci.

[CR6] Berghem LER, Petterson LG, Axiofredrikson UB (1976). The mechanism of enzymatic cellulase degradation. Isolation and some properties of a β –glucosidase from trichodermaviride (edited by Berghem, L.E.R, and Petterson, L.G). Eur J Biochem.

[CR7] Bisaria VS, Ghose TK (1981). Biodegradation of cellulosic materials substrates, microorganisms, enzymes and products. Enzymes Microb Technol.

[CR8] Bradbury MG, Egan SV, Bradbury JH (1999). Determination of all Forms of Cyanogens in Cassava Roots and Cassava Products Using Picrate Paper Kits. J Sci Food Agr.

[CR9] Bukoye MA (2001). Use of Cellulosic wastes for Cellulase Production by Aspergillus niger-CS9 and Penicillium digitatum-YF50.M.Sc Dissertation.

[CR10] Chahal DS, Kennedy (1992). Bioconversion of polysaccharides of lignocelluloses and simultaneous degradation of lignin. Lignocellulosics: Science Technology Development and Use.

[CR11] Christopher –Berg FOL (2006). The world ethanol industry shows no sign of slowing down. The Foss group Journal of Technology for Food, Diary and Agricultural analyses.

[CR12] Dipeolu MA, Eruvbetin D, Williams TJ (1996). Indigenious chicken rearing under village conditions. Int J Anim Sci.

[CR13] FAO: *Food and Agricultural Organization. World food production, Geneva FAO nutritional meeting*. 1986, 210. [*Report series NO. 52*]

[CR14] (2010). GHANA- feasibility study on the use of cassava waste to produce energy.

[CR15] Hafeni S, Mpofu IDT, Petrus P (2013). The potential of pearl millet and water melon seeds as cheap alternative ingredients in Namibian poultry feeds. Agr Sci Res J.

[CR16] Kayode RM (2009). Suitability of Mango (Mangiferaindica) fermented kernel as animal feed supplement.

[CR17] Larry EN (1993). Broiler feeding and management. Poultry Int.

[CR18] Lawal TE, Iyayi EA, Aderemi FA (2005). Biodegradation of groundnut pod with extracted enzymes from some isolated tropical fungi: Growth resources and carcass quality of broilers finisher birds. Proceedings of Annual Conf Anim Sci Ass of Nig (ASAN).

[CR19] McGinnins MR (1980). Laboratory handbook of medical mycology.

[CR20] Moo-young M, Lamptey J, Glick B, Bungay H (1987). Biomass conversion technology.

[CR21] Muhammad NO, Oloyede OB (2009). Haematological parameters of broiler chicks fed Aspergillus niger - Fermented Terminalia catappa seed meal-based diet. Global J Biotechnol Biochem.

[CR22] (1984). Nutrient Requirement of poultry.

[CR23] Nworgu FC, Egbunike GN, Ogundola FI (2000). Performance and nitrogen utilization of broilers fed full fat extruded soybean meal and full fat soybean. Tropical Animal Production Investment.

[CR24] Oruwari BM, Sese BT, Mgbere OO (1995). The effect of whole palm kernel on broiler performance and production cost: energy protein ratio. Int J Anim Sci.

[CR25] Panda T (1989). Simulation of shake flask conditions in a bioreactor for the biosynthesis of cellulases and xylanases by a mixed culture of *Trichodermareesi* D 1–6 and *Aspergilluswentil*pt- 2804 process. Biochemistry.

[CR26] Sani A, Awe FA, Akinyanju JA (1992). Amylase synthesis in Aspergillusnigerand Aspergillusflavus grown on cassava peel. J Ind Microbiol.

[CR27] Sabiiti EN (2011). Utilizing agricultural waste to conserve food security and conserve the environment. Afr J Food Nutr Sci.

[CR28] Sibbald IR (1982). Measurement of bioavaialable energy in poultry feedstuff. A review. Can J Anim Sci.

[CR29] Steiner J, Socha C, Enzaguirre J (1994). Culture condition for enhanced cellulase production by a native strain of Penicilliumpurpurogenum. World J Microbiol Biotechnol.

[CR30] Tewe OO, Egbunike GN, Hahn SK, Reynolds L, Egbunike GN (2007). Utilization of cassava in non ruminant feeding. Cassava as livestock feed in Africa.

[CR31] Thackie AM, Flenscher JE (1995). Nutritive value of wild sorghum fortified with leucaena (*Leucaena leucocephala* Wh. Lam.). Bulleon Animal Health, Africa.

